# Prevalence of Verbal Abuse Among Doctors in Tertiary Care Hospital

**DOI:** 10.31729/jnma.4762

**Published:** 2019-12-31

**Authors:** Kalpana Silwal, Sarala Joshi

**Affiliations:** 1 Department of Nursing, Chitwan Medical College and Teaching Hospital, Bharatpur, Chitwan, Nepal; 2 Om Health Institute, Nursing College, Kathmandu, Nepal

**Keywords:** *abuse*, *doctors*, *workplace*

## Abstract

**Introduction:**

Verbal abuse is the act of forcefully criticizing, insulting or denouncing another person. Verbal abuse can be devastating to doctors and may cause long lasting emotional and psychological damage. This study aims to find the prevalence of verbal abuse among doctors in tertiary care hospital.

**Methods:**

This descriptive cross-sectional study was conducted among doctors in a tertiary care hospitals, Chitwan from January to July, 2019 after taking ethical approval. Convenience sampling was done. Self administered questionnaire was distributed and data was collected. Point estimate at 95% CI was done for binary data along with frequency and proportion. Data were entered and calculations were done in Microsoft excel.

**Results:**

Verbal abuse was found among 80 (33.3%) respondents at 95% Confidence Interval (27.51-39.09%) and most 51 (63.8%) of perpetrators were relatives of the patients. Most 38 (47.5%) of the doctors were often worried in workplace. Incident of the verbal abuse was more 35 (43.6%) in morning and least 14 (17.4%) in night. Most 22 (27.5%) of the doctors did not take any action for incident although most 42 (52.5 %) of the doctors were encouraged by colleagues to take action.

**Conclusions:**

Prevalence of verbral abuse among the doctors were found out to be similar as the previous studies conducted in similar settings. This study has shown that doctors were frequently verbal abused by patient's relatives and were abused mostly in morning shift and were often worried in workplace.

## INTRODUCTION

Verbal abuse has been known as a significant issue Verbal abuse against doctors can affect the work performance and productivity in hospital.^1^

The prevalence of workplace violence in the last 12 months was found to be 63.41%, whereas the lifetime prevalence was found to be 78.05%.^2^ Violence against doctors is increasing in Nepal and will continue to surge until the effective measures to improve healthcare in our country.^3^ However, this issue is under researched and little evidence exists so the researcher interest to do study on this research problem.

Maximum (87.3%) of the reported cases were of verbal violence while 8.6% of the cases were of physical violence.^4^ Higher rates of burnout, low self-esteem and self-destructive aggression are main symptoms in health care professions.^5^

The present study aims to find the prevalence of verbal abuse among doctors in a tertiary care hospital.

## METHODS

This descriptive cross-sectional study was conducted in two tertiary care hospitals: Bharatpur hospital and Chitwan Medical College and Teaching Hospital, over a period of January to July 2019. Ethical approval was taken from Ethical Review Board of National Health Research Council and also from Chitwan Medical College and Teaching Hospital and Bharatpur Hospital.

During the study 276 questionnaires were distributed, 255 were returned and 15 were discarded due to incomplete information for the study.

Convenience sampling was done and sample size was calculated using the formula:

$$\begin{array}{ll} \text{n} & ={\text{Z}}^{\text{2}}\times \text{(p}\times \text{q)}/{\text{e}}^{\text{2}}\\ & =\text{1}{\text{.96}}^{\text{2}}\times \text{0.3}\times (1-\text{0.3)}/\text{0}{\text{.06}}^{\text{2}}\\ & =224 \end{array}$$

where,

n= required sample size
p= prevalence the burden of verbal abuse among doctors (30%, educated guess)
q= 1-p
e= margin of error, 6%
Z= 1.96 at 95 % CI

The total sample size calculated was 224 and after adding non-response rate as 7%, the total sample size taken was 240. The data was collected in a selfadministered questionnaire and analyzed by Statistical Package for Social Sciences 20 version. Point estimate at 95% CI was done for binary data along with frequency and proportion.

## RESULTS

Out of 240 study participants, verbal abuse was found in 80 (33.3%) participants at 95 % CI (27.51-39.09) (Table 1). Thirty eight (47.5%) were found to be often worried and 5 (6.3%) were never worried. Out of total, 42 (52.5%) respondents were encouraged by colleague to take actions for the verbal abuse but 22 (27.5%) respondents did not take any actions (Table 1).

**Table 1:** Distribution of Verbally abused in workplace.

Statement	n (%)
Verbally abused (Yes)	80 (33.3)
Statement	
verbally abused in the last 12 months (n=80)	
always worried	20 (25)
often worried	38 (47.5)
sometimes worried	9 (11.2)
seldom worried	8 (10)
Never worried	5 (6.3)

Majority 170 (70.8%) of the respondents were verbally abused for 6-12 months and 1 (0.4%) of the respondents were verbally abused for 37-48 months (Table 2). Out of total abused, 35 (43.6%) faced verbral abuse on morning time followed by evening and night.

**Table 2:** Respondents’ work experience and time of Incident of verbal abuse.

Time of verbral abuse (n=80)	n (%)
Morning	35 (43.5)
Evening	31 (39.0)
Night	14(17.5)

Perpetrators in the last 12 months,majority 51 (63.8%) perpetrators were relatives of the client or patient (Table 3). Least 14 (17.5%) perpetrator were supervisors or managers.

**Table 3:** Distribution of perpetrators in the last 12 months.

Perpetrators of the verbal abuse (n=80)	n (%)
patient/client	15 (18.8)
relatives of the patient/client	51 (63.8)
management/supervisor	14 (17.5)

Sociodemographic characteristics of the participants is shown The mean age of the participants was 27.82±4.52 years and 215 (89.5%) were the age of 21-25 years. Majority of the participants; 153 (63.7%) were not married and 213 (88.8%) were full time workers (Table 4).

**Table 4:** Respondents’ socio-demographic characteristics.

Age of respondents (years	n (%)
21-25	215 (89.5)
26-30	22 (9.2)
31-35	3 (1.3)
Total	240 (100)

## DISCUSSION

In our study, verbal abuse was found among 80 (33.3 %) respondents. A study conducted in New Delhi , India had found that verbal violence was the most common type of violence, 62 (87.3%).^4^ According to the data of the Bureau of Labour Statistics (BLS), USA reported that prevalence of workplace violence among physicians to be 56%-75%^6^ in 2014 in Manipur found that 78% of doctors had experienced some form of violence.^7^ Our study has shown that most of perpetrators 51 (63.8%) were relatives of the patients. Study in India also showed the similar finding i.e. patients and their relatives are the most common perpetrators.^6^ Other study also reported that in most of the cases of violence, the perpetrators were either patients or their relatives. Either patients or their attendants were the main perpetrators of violence which was 84.5%.^4^ Our study showed that the incidence of the verbal abuse was more 35 (43.6%) in morning and least 14 (17.4%) in night. Similar finding was by a study which showed that maximum events of the verbal abuse took place in morning than evening and least night shifts.^4^ Another study found that verbal abuse incidence occurred especially in evening and night time which is contradict of this study.^7^ In this study, we found out that most 38 (47.5%) of the respondents were often worried in workplace. One of the study reported that the increasing incidences of violence against doctors in their workplaces is the important reason for stress among these healthcare workers. Maximum (87.3%) of the reported cases were of verbal violence.^4^ Other study also showed that higher rates of burnout, low self-esteem and self-destructive aggression are among the violence in the health care system.^5^

A study concluded that verbal abuse obviously impact on the effectiveness of health systems, especially in developing countries which should minimize and should prevented as much as possible.^6^ Other study had suggested that organizations must adopt zero-tolerance policies and training and in-service education should be given to prevent incidence of the verbal abuse.^7^

## CONCLUSIONS

Prevalence of verbral abuse among the doctors were found out to be similar as the previous studies conducted in similar settings. This study has shown that doctors were frequently verbal abused by patient's relatives and were abused mostly in morning shift and were often worried in workplace. Furthermore, for the nation's parlous state of healthcare to improve, much emphasis should be placed on ensuring to prevent abuse in the workplace. So necessary that certain strategies are plan to guarantee the safety of health care providers in every health care institutions in Nepal.
